# Helping people with psychosis with a low-cost intervention DIALOG+: protocol for the economic evaluation in a randomised control trial in India and Pakistan

**DOI:** 10.1136/bmjopen-2023-080737

**Published:** 2025-01-08

**Authors:** Ashar Muhammad Malik, Renata Peppl, Sana Zehra Zehra Sajun, Onaiza Qureshi, Krishna Priya, Hufsa Sarwar, Padmavati Ramachandran, Lakshmi Venkatraman, Sara Evans-Lacko, Victoria Jane Bird

**Affiliations:** 1 Community Health Sciences, The Aga Khan University Faculty of Health Sciences, Karachi, Pakistan; 2 Faculty of Arts and Sciences, The Aga Khan University, Karachi, Pakistan; 3 Unit of Social and Community Psychiatry, Queen Mary University of London Faculty of Medicine and Dentistry, London, UK; 4 Interactive Research & Development, Karachi, Pakistan; 5 Schizophrenia Research Foundation, Chennai, Tamil Nadu, India; 6 Mental Health, Interactive Research & Development, Karachi, Pakistan; 7 Psychiatry, Schizophrenia Research Foundation, Chennai, Tamil Nadu, India; 8 Personal Social Services Research Unit, London School of Economics and Political Science, London, UK; 9 Unit for Social and Community Psychiatry, Queen Mary University of London Faculty of Medicine and Dentistry, London, UK

**Keywords:** HEALTH ECONOMICS, Schizophrenia & psychotic disorders, Randomized Controlled Trial, PUBLIC HEALTH, Quality of Life, Health Care Costs

## Abstract

**Background:**

Approximately 69%–89% of people with severe mental illnesses, particularly psychosis, experience a treatment gap in low- and middle-income countries (LMICs) due to factors such as low public spending on health and weak healthcare systems. The PIECEs project aims to assess the effectiveness and cost-effectiveness of a solution-focused resource-oriented approach (DIALOG+) for improving the quality of life and mental well-being of people with psychosis in India and Pakistan.

**Methods:**

The research design of this analysis is an economic evaluation piggybacked on the PIECEs randomised control trial to test the feasibility of DIALOG+ in India and Pakistan. It implies a cost-utility analysis with a health system perspective. The costs include the cost of the intervention, the cost of healthcare providers and the cost to the household. The primary outcome will be quality-adjusted life years. Incremental cost, incremental effectiveness and incremental cost-effectiveness ratios will be calculated using linear regression models with a hierarchical data structure. A probabilistic sensitivity analysis will be carried out to test for the uncertainty surrounding the estimates of cost-effectiveness.

**Discussion:**

This study will provide evidence of a patient-centred approach to improve the quality of community-based care for people with psychosis in India and Pakistan. The economic evaluation will support efforts to scale up low-cost healthcare interventions such as DIALOG+ to rural and unserved areas, which is otherwise challenging in the resource-constrained health systems in many LMICs.

**Conclusion:**

The evidence on the cost-effectiveness of DIALOG+ will contribute to efforts to improve community-based care and the quality of life for millions of people suffering from mental health problems in India and Pakistan who experience psychosis.

**Ethics and dissemination:**

This study is approved by the Queen Mary Ethics of Research Committee (UK), Institutional Ethics Committee of SCARF (India), IRD’s Independent Institutional Review Board (IRD_IRB_2021_01_005) (Pakistan), Karawan-e-Hayat Management Committee (Institutional Approval) (Pakistan), Jinnah Postgraduate Medical Centre Research Committee (NO.F.2-81/2021-GENL/60224/JPMC) (Pakistan), Aga Khan’s Ethics Research Committee (2021-5933-17533) (Pakistan) and National Bio-Ethics Committee, Pakistan (Ref: No.4–87/NBC-774/22/2037 Date: 17 May 2022).

The findings of this research will be widely disseminated through research publications and engagement with the communities and the healthcare providers in the public and not-for-profit sectors.

**Trial registration number:**

ISRCTN13022816.

**STRENGTHS AND LIMITATIONS OF THIS STUDY**

- This will be among the first cost-utility analyses on psychosis management in randomised control trials in low- and low-middle-income countries.
- The economic evaluation will be carried out jointly and separately for each country.
- Including the public sector and the not-for-profit sector will improve the generalisability of the results.
- Due to ethical considerations, the intervention arm, DIALOG+ is being compared with an active control rather than routine practice.
- Using a health system perspective will avoid some non-healthcare costs, though few exist.

## Introduction

 Mental illnesses are among the top 10 leading causes of the global burden of diseases.^1^ Management of mental illnesses is a major policy imperative in high-income countries. In low- and low-middle-income countries (LMICs), priority setting in the health sector focuses on acute and life-saving care and is often supplemented by the development assistance to LMICs prioritising the healthcare needs of women and children. Neglecting mental illnesses and other non-communicable diseases in public spending on health in LMICs shifts the cost to the household’s resources and increases their risk of financial catastrophic costs compared with communicable diseases (risk difference=1.71%; 95% CI 0.75–2.67).^2^ There is a dearth of mental health services in LMICs such as India and Pakistan—approximately one psychiatrist for half a million population. Access to mental health is further compromised as mental services are concentrated in urban areas. Due to such factors, approximately 70%–80% of people with severe mental illness experience a treatment gap in LMICs.^3 4^ Finding a low-cost solution to improve treatment for mental illness is a crucial aspect of health sciences research in severely resource-constrained health systems in LMICs.

The PIECEs project aims to test the effectiveness and cost-effectiveness of a solution-focused, resource-orientated and patient-centred approach (DIALOG+) for improving the quality of life of people with psychosis in India and Pakistan. The project is funded by a grant from the National Institute of Health and Care Research, UK. The project comprises five work packages being implemented over 4 years. Work package 2 of the project is a cluster randomised control trial (RCT) in India and Pakistan.^5^ The RCT evaluates the effectiveness of DIALOG+: a computer-mediated intervention developed by the Unit of Social Psychology, Queen Mary University of London, and has been tested in six European countries.^6^

The PIECEs RCT is being conducted in India and Pakistan. Details of the PIECEs project and the protocol for the RCT are published elsewhere.^7 8^ In this paper, we present the protocol for piggybacking economic evaluation with the RCT. We anticipate that this information is relevant to the academic audience of economic evaluation in clinical trials in LMICs. In the backdrop of the facts, resource allocation follows historical budgeting, and reimbursement policies follow expert opinions;^9^ the results of this study will contribute towards the efforts to promote the use of economic evidence in medical decision-making in LMICs.

## Methods

### PIECEs RCT

**Figure 1:**
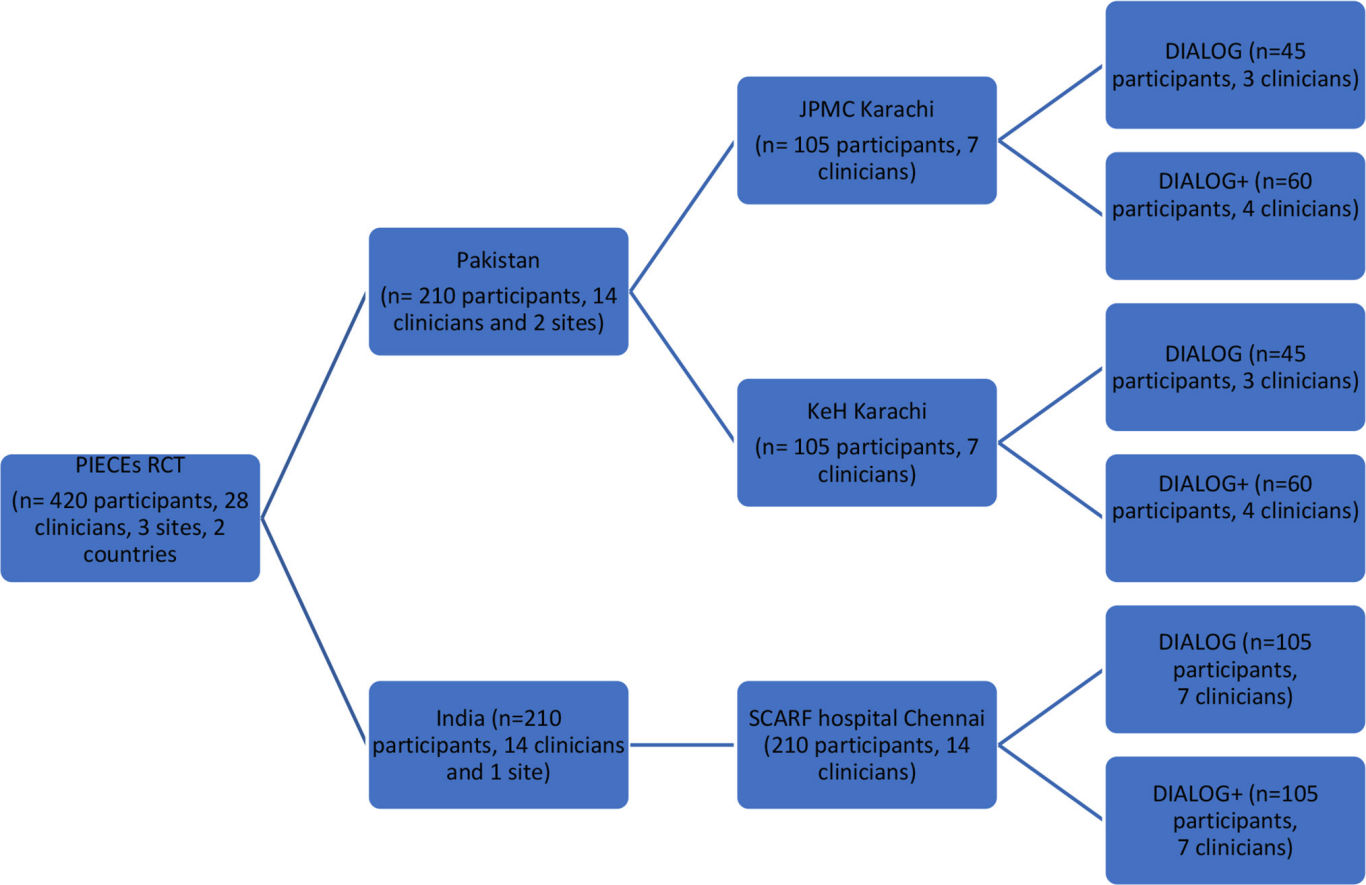
DIALOG+ intervention is compared with an active control DIALOG in the PIECEs RCT in the field sites: Jinnah Post Graduate Medical Centre (JPMC), Karachi; Karwan-e-Hayat not-for-profit hospital (KeH), Karachi; and Schizophrenia Foundation of India Hospital (SCARF), Chennai. Figure Design of the Clustered Randomised Control Trial of the NIHR-funded PIECEs research project.

The PIECEs RCT has three objectives: (1) to test the feasibility of DIALOG+ (treatment) with active control (DIALOG scale only) for improving quality of life and clinical and social outcomes, (2) to conduct an economic evaluation of the intervention and (3) to understand the experience and acceptability of DIALOG+ within routine services in India and Pakistan. The PIECEs RCT is being conducted (1 September 2020 to 28 February 2025) at three sites in Chennai, India, and Karachi, Pakistan. In Karachi, the two sites are Jinnah Post Graduate Medical Centre, a public-sector teaching hospital, and Karwan-e-Hayat, a not-for-profit outpatient psychiatric clinic. In Chennai, the field site is Schizophrenia Research Foundation Hospital, a not-for-profit teaching hospital. The sample size (n=420) of the RCT is powered to draw conclusions at country levels in India and Pakistan (figure 1).

### Ethics and dissemination

This study is approved by the Queen Mary Ethics of Research Committee (UK), Institutional Ethics Committee of SCARF (India), IRD’s Independent Institutional Review Board (IRD_IRB_2021_01_005) (Pakistan), Karawan-e-Hayat Management Committee (Institutional Approval) (Pakistan), Jinnah Postgraduate Medical Centre Research Committee (NO.F.2-81/2021-GENL/60224/JPMC) (Pakistan), Aga Khan’s Ethics Research Committee (2021-5933-17533) (Pakistan) and National Bio-Ethics Committee, Pakistan (Ref: No.4–87/NBC-774/22/2037 Date: 17 May 2022).

The findings of this research will be widely disseminated through research publications and engagement with the communities and the healthcare providers in the public and not-for-profit sectors.

### Patient and public involvement

At the initiation of the study, both partner countries set up local Lived Experience Advisory Panels (LEAP), which are formed of people who have recent and relevant lived experience of mental health challenges, carers of people who have recent and relevant experience of mental health challenges and clinicians/facilities staff who also share these lived experiences. The LEAP provides ongoing feedback to improve the relevance, practicality and influence of the research. This is done by drawing on their own experience of and local knowledge about the study sites to advise and steer the researchers, advising on and assisting in the recruitment and participation of people who use local services or participate in the research and make contributions to any events at the study sites and will also be involved in the dissemination of study findings.

### Intervention

DIALOG+ is a psychosocial tool to strengthen the quality of person-centred care by improving the therapeutic alliance between care providers and mental health service users. DIALOG+ is studied extensively in European, South American and African contexts.^7 10 11^ There is limited evidence for its feasibility and acceptability in low-resource settings in South Asia.

In the following subsections of the methods, we describe the protocols for the economic evaluation piggybacking on the PIECEs RCT. We follow the guidelines on reporting the economic evaluation of healthcare programmes developed by the task force on good research practice in the economic evaluation of the International Society for Pharmacoeconomics and Outcomes Research and the checklist for reporting the economic evaluation recommended by Drummond *et al*.^12 13^ These documents provide a step-by-step guide for researchers and academics on methods to conduct economic evaluation. Moreover, these documents promote the standardisation of methodology for conducting and reporting economic evaluations at a global level.

### Aim and objective

The economic evaluation component of the PIECEs RCT aims to provide evidence of the economic evaluation of DIALOG+ in India and Pakistan. This will include a detailed costing exercise and application of locally generated values sets of EQ-5D to estimate quality-adjusted life Years (QALYs).

### Perspective on economic evaluation

The perspective taken within the economic evaluation is that of the health system, including the patient and their family. This perspective is consistent with the objectives and scope of our study, which aims to evaluate a low-cost intervention that can be integrated into existing health systems and delivered by non-specialist health workers. Other than healthcare, there are limited formal social institutions that cater to the needs of people with severe mental illness. In India and Pakistan, social welfare systems are weak and nearly non-existent for people with mental illnesses. Mental health is a low priority in the health sector as demonstrated by priority setting at national and subnational levels.^14^ Moreover, community-based approaches for the early management of mental illnesses are in their infancy in the Indian subcontinent.^15 16^ Lastly, the share of social health protection in healthcare financing in India and Pakistan is negligible: 2% and 1% of current health expenditure, respectively.^17^

### Type of Analysis

We will carry out a cost-utility analysis (CUA) and cost-effectiveness analysis (CEA). In both cases, e-costs are estimated in monetary terms. Outcomes in the CUA are estimated in quality and length of life, and in the case of CEA, outcomes are reported in natural units using the Manchester Short Assessment of Quality of Life (table 1). Costs will be estimated for patients and health systems. The main outcome of the CUA will be QALYs. We chose QALY as an outcome measure because it captures the impact of the intervention in physical and mental health domains and across quality and length of life aspects. CUA using QALYs allows a comparison of interventions with any intervention in medical care. In the case of CEA, the comparison of interventions is limited within the mental health domain. We will use common methods for estimating costs, estimating outcomes, reporting results and dealing with uncertainty in both types of analyses.

**Table 1:** Data collection tools used in the economic evaluation in the clustered randomised control trial of the PIECEs research project

Type	Cost centre	Instrument	Unit/data source	Data collector	Frequency of data collection	Data storage software
Resource use	Patient and caregiver costs	CSRI	Patient	Researcher	Three times (baseline, 6 months and 12 months	RedCap
	Health facility costs	TMS data collection sheet	Facility	Researcher	Two times (baseline, 6 months)	Manual/Microsoft Excel
		Facility data collection questionnaire	Facility	Researcher	Once	Manual/Microsoft Excel
	RCT cost	Quarterly Expenditure Report and Expense Claims forms	International Research Management QMUL	Economic analyst	Once	Manual/ Microsoft Excel
Outcomes	Quality length of life	EQ-5D-5L	Patient	Researcher	Three times (baseline, 6 months and 12 months)	RedCap
	Mean item score	MANSA	Patient	Researcher	Three times (baseline, 6 months and 12 months)	RedCap
Socio-demographic and other stratifies	Basic demographic data, including gender, age, ethnicity, marital status and education	Basic demographic form	Patient	Researcher	Once (baseline)	RedCap

Research Electronic Data Capture (RedCap) is a data collection and management software ofdeveloped by Vanderbilt University, United States of AmericaSA.

CSRIClient Service Receipt InventoryMANSAManchester Short Assessment of Quality of LifeRCTrandomised control trialTMStime and motion study

### Choice of comparators

The PIECEs RCT includes two arms: the intervention arm, DIALOG+, and the active control arm, DIALOG. Participants in the intervention arm receive treatment in two steps during their routine consultations with clinicians. First, a structured patient assessment (DIALOG) covering satisfaction (Likert scale of 1–7) in 11 domains (8 life domains and 3 treatment domains). Next is a four-step solution-focused therapy (+ component) approach to address patient concerns. The intervention is delivered during routine consultations and makes use of a tablet or smartphone. To control for the addition of a tablet computer in the consultation and for repeated quality-of-life assessments, patients in the control arm will complete the DIALOG scale at the end of each session excluding the four-step solution-focused component. Patients in both arms will continue to receive standard treatment, including routine meetings with clinicians.

### Time horizon

The time horizon of the economic evaluation will be 1 year, including the RCT implementation period of 6 months and a follow-up of 6 months.

### Costs and resource use

Costs will be estimated for resources provided to the intervention and active control arms. Three cost centres include (a) the resources associated with the health facilities such as the use of building space, utilities and supplies, and time costs of mental health professionals and medical and support staff providing care and (b) resources of the patient and their caregiver for seeking healthcare, including travelling, accommodation and productivity losses, and (c) the resources associated with running the RCT, such as staff training, incentives to the clinicians and tablets for data collection.

Health facility resources for the provision of healthcare to the participants in the PIECEs RCT include resources provided by the health facility including the time cost of health personnel, use of consulting clinics and waiting areas, instruments, equipment and supplies during the consultation process at the respective health facility. The data for health facility resource use will be collected in two separate studies: (a) time and motion study (TMS) and (b) health facility-level data collection.

- The data for the TMS will be collected from a subsample of the RCT participants (n=180). The sample is stratified by health facility, gender and interval of data collection (60 participants—20 participants at each data collection point, baseline, 6 months and 12 months). A data collector unblinded to RCT participants will record the time on a stopwatch spent by the RCT participants starting from the registration desk to the time they leave the consulting clinic. The difference between the start and end times of the consultation will provide the estimated time spent at the health facility for the patient visit.
- The health facility level data will be collected from the financial and administrative records of health facilities for the financial year 2022–2023. This will include expenditure data of health facility-level resources used for the provision of health services, including salaries of medical, nursing and support staff, expenditure on fixed assets, utilities and medical and other supplies. A bespoke health facility collection tool was developed in Microsoft Excel and shared with each health facility to complete with relevant information. The data collected will be used to estimate the health facility cost of a patient visit to psychiatry clinics.

The patient and caregiver resources include the cost of travel to the health facility, productivity losses associated with their healthcare consultation and the out-of-pocket payment on doctor’s fees, lab tests and medicines. The data for patients and caregiver resources will be extracted from three questionnaires: a socio-economic and demographic questionnaire, a visit information questionnaire and a modified Client Service Receipt Inventory (CSRI). The socio-economic and demographic questionnaire will collect employment information, and the CSRI will collect information on earnings to estimate productivity losses in combination with data from the visit information questionnaire (ie, number of visits and accompanying persons) and the CSRI (to estimate the number of working days lost). Out-of-pocket health expenditure will also be extracted from the modified CSRI. The frequency of this data collection is baseline, 6 months and 12 months (table 1). These forms are adapted for the local context of India and Pakistan, for example, occupation codes, modes of travel and types of health providers. All data collection tools used in the RCT are translated into local languages: Urdu for Pakistan and Tamil for India.

The RCT resources include the provision of tablet computers, incentives to healthcare providers and their training on using the DIALOG+ application in intervention and active control arms of the RCT. We will exclude research costs associated with running the trial, collecting data and outcome assessment.

The data on costs of intervention and active control of the RCT will be extracted from the quarterly expense reports of the implementation partners of the RCT and the expenditure statement of the PIECEs grant held with the Unit of Social Psychiatry of the Queen Mary University of London. A summary of data collection tools and frequency of data collection is provided in table 1. All data will be collected by trained researchers.

The costs of three types of resources—patient/caregiver, health facility and the RCT resources—will be aggregated at the patient level. To generalise healthcare providers’ costs obtained from the subsample of the PIECEs RCT participants, we will use semiparametric bootstrapping methods to account for noise generated by clustering of demographic features of the participants and the type of health facility.

### Currency, price date and conversion

The local CEA will be reported in Indian rupees (INR) and Pakistan rupees (PKR) for local audiences in India and Pakistan, respectively. All resource use will be reported in the prices of 2022–2023. The costs of previous years will be inflated to current years using the official inflation rates of India and Pakistan. The pooled CEA will be reported in US dollars (USD) and Great Britain pounds (GBP). Costs will be converted to an average of yearly averages of the USD and GBP conversion rates for INR and PKR in 2022 and 2023.

### Outcome

We will use the EQ-5D-5L to assess health-related quality of life at baseline, 6 months and 12 months. The EQ-5D-5L is a generic instrument for measuring the quality of life across five dimensions: mobility, self-care, usual activities, pain/discomfort and anxiety/depression. Each dimension has five levels: no problems, slight problems, moderate problems, severe problems and extreme problems. The Urdu and Tamil versions of EQ-5D-5L are obtained from the EUROQOL group. These data will be used to estimate QALYs by using the population preferences (values set) for the health states mentioned above.^18^ The value set of EQ-5D-5L developed by Jyani *et al* for India will be used to estimate QALYs for the Indian field site of the PIECEs RCT. This value set is developed using a time trade-off from a population-representative sample (n=3548) for geographical representation, including the Tamil region (the PIECEs RCT research site).^19^ In Pakistan, the data collection of values set on EQ-5D-3L has been completed. By the time the PIECEs RCT data collection is complete, we anticipate that the value set of EQ-5D-3L will be available in the public domain (Email Communications with EUROQOL, 13 December 2022). We will use the methods reviewed by van Hout *et al* to obtain a value set for EQ-5D five levels using the three-level values set of EQ-5D being generated for Pakistan.^20^

### Analysis and reporting results

We will conduct a primary analysis at the country level for India and Pakistan separately, using local currencies (INR and PKR, respectively) and country-specific value sets for EQ-5D-5L to estimate QALYs. We will also conduct a secondary pooled analysis for both countries combined, using USD and GBP and a common value set for EQ-5D-5L. We will report the results of the analysis using the most recent recommendation of the Taskforce on consolidated health economic evaluation reporting standards (CHEERS).^10^ The economic evaluation results will be reported as incremental cost-effectiveness ratios (ICERs). We will estimate the ICERs as differences in costs divided by differences in QALYs between DIALOG+ and DIALOG.^11^ Similarly, the incremental QALYs will be estimated as the difference in QALYs of the patient treated with DIALOG+ and QALYs in active control DIALOG, that is,

ICER=Cost (Dialog+)–Cost (Dialog)/QALY (Dialog+)–QALY (Dialog).

We will explore the degree of clustering due to nesting in data across clinicians by estimating the intracluster correlation coefficient. Further, we will explore using multilevel and/or multiple linear regression models to find differences in differential cost in two arms of the PIECEs RCT. In India, such levels in regression models will include RCT participants clustered among clinicians.

We will explore the acceptability of DIALOG+ in the cost-effectiveness plan. We will use the locally adopted threshold values of a QALY to determine if DILOG+ is worth adopting for psychosis management in India and Pakistan. A literature search will be carried out to find QALY threshold values. Alternatively, we will use the recommendations of the WHO to value a disability-adjusted life year by three times the per capita gross domestic product of the respective country.^21^

From the pooled economic evaluation for India and Pakistan, we will account for multiple sources of variability between the two countries drawing probabilities by varying assumptions on healthcare financing, hospital reimbursement and formulary decisions, health-seeking behaviour and incentives to healthcare providers.^22^ We will explore appropriate models to account for clustering within countries/jurisdictions. In the robustness check, we will compare estimates on incremental cost, incremental effects and ICERs from ordinary least square linear regression with fixed effects of health facility and country indicators and a hierarchical linear model accounting for nesting of data at country, health facility levels and patient-specific factors such as gender.^23^

### Characterising uncertainty

One-way and two-way probabilistic sensitivity analyses will be used to test for the robustness of the reported mean costs, incremental costs, incremental QALYs and ICERs.^24^ Uncertainty will arise from multiple sources, for example, the assumptions used for estimating costs of healthcare facilities, using generalising EQ-5D-3L value set for EQ-5D-5L, etc. To deal with missing data or censored data, we will rely on the statistical methods used for the analysis of the RCT data. Bayesian and frequentist statistical techniques will be used to understand the influence of skewed distribution and correlation in cost and/or outcomes data, for example, regression approaches and bootstrapping of cost, outcomes in pairs or Bayesian bivariate models, respectively.^23^

All analysis will be carried out in Microsoft Excel 365 and STATA V.16 for Windows 11 licensed from Microsoft Corporation Inc. and Stata Corporation Inc., respectively.

## Discussion

To the best of our knowledge, the economic evaluation component of the PIECEs RCT will be among the first lines of evidence on managing psychosis with a cost-effective intervention in India and Pakistan that is relevant to other LMICs including South Asia.

We provide appropriate methodologies to the research community on economic evaluation in clinical trials in LMICs, with limited resources to finance health services to the population. By doing so, we will contribute to efforts on cost considerations or using economic evaluation evidence in setting priorities in the health sector in LMICs, which are otherwise occasionally considered partly due to a shortage of expertise in the field of health economics and the fact that costs tariffs of health services are not a mandatory requirement in medical decision-making. Our methodology for economic evaluation in clinical trials in LMICs proposes protocols for additional data collection and analysis to estimate costs tariffs that are otherwise readily available in developed countries.^25^

The breakdown of the costs by the types of healthcare providers will facilitate efforts to use costing as a management tool in hospital management practices. For example, with readily available costs of services, hospital managers can determine the budget impact of the management of psychosis with DIALOG+. At the national level, such estimates will be included while designing the essential packages of services to achieve the Universal Health Coverage envisaged in the Sustainable Development Goals.^26^

In this paper, we provide the protocol for piggybacking the economic evaluation on the PIECEs RCT. Methods related to data collection, data cleaning, imputation and methods and approaches for dealing with missing and censoring data are provided in the protocols of RCT and the statistical analysis plan for the PIECEs RCT. Our analysis is limited in scope, as instead of routine care, we compare intervention with active control. Lastly, our analysis uses a health system perspective avoiding CHEER recommendations of using a broader social perspective in economic evaluation.

## Conclusion

Economic evaluation is the framework of evidence-based decision-making in healthcare. Findings from economic evaluation will aid the efforts to use economic evidence in priority settings at the clinical level or priority settings in reimbursement policies or the national level. Methods proposed in this paper will encourage the research community to piggyback economic evaluation in ongoing or planned clinical trials in LMICs. Our findings will promote local-level decision-making and adoption of DIALOG+ as a cost-effective as well as low-cost intervention for the management of psychosis in India and Pakistan.

## References

[1] GBD (2022). Global, regional, and national burden of 12 mental disorders in 204 countries and territories, 1990–2019: a systematic analysis for the Global Burden of Disease Study 2019. Lancet Psychiatry.

[2] Murphy A, Palafox B, Walli-Attaei M (2020). The household economic burden of non-communicable diseases in 18 countries. BMJ Glob Health.

[3] Evans-Lacko S, Aguilar-Gaxiola S, Al-Hamzawi A (2018). Socio-economic variations in the mental health treatment gap for people with anxiety, mood, and substance use disorders: results from the WHO World Mental Health (WMH) surveys. Psychol Med.

[4] Demyttenaere K, Bruffaerts R, Posada-Villa J (2004). Prevalence, severity, and unmet need for treatment of mental disorders in the World Health Organization World Mental Health Surveys. JAMA.

[5] Improving community-based mental health care in India and Pakistan. https://piecesresearch.com.

[6] Priebe S, McCabe R, Bullenkamp J (2007). Structured patient-clinician communication and 1-year outcome in community mental healthcare: cluster randomised controlled trial. Br J Psychiatry.

[7] Bird VJ, Sajun SZ, Peppl R (2023). Assessing the effectiveness and cost-effectiveness of a solution-focused resource-orientated approach (DIALOG+) to improving the quality of life for people with psychosis in India and Pakistan-a cluster RCT. Trials.

[8] Bird JV (2017). Improving outcomes for people with psychosis in Pakistan and India – enhancing the effectiveness of community-based care (PIECES). Protocol.

[9] Thatte U, Hussain S, de Rosas-Valera M (2009). Evidence-based decision on medical technologies in Asia Pacific: experiences from India, Malaysia, Philippines, and Pakistan. Value Health.

[10] Priebe S, Kelley L, Golden E (2013). Effectiveness of structured patient-clinician communication with a solution focused approach (DIALOG+) in community treatment of patients with psychosis--a cluster randomised controlled trial. BMC Psychiatry.

[11] Birabwa-Oketcho H, Nakasujja N, Alinaitwe R (2023). The effectiveness of a solution-focused approach (DIALOG+) for patients with severe mental illness and epilepsy in Uganda: A randomised controlled trial. Psychiatry Res Commun.

[12] Husereau D, Drummond M, Augustovski F (2022). Consolidated Health Economic Evaluation Reporting Standards (CHEERS) 2022 Explanation and Elaboration: A Report of the ISPOR CHEERS II Good Practices Task Force. V H.

[13] Drummond MF, Sculpher MJ, Claxton K (2015). Methods for the Economic Evaluation of Health Care Programmes.

[14] Alvi MH, Ashraf T, Kiran T (2023). Economic burden of mental illness in Pakistan: an estimation for the year 2020 from existing evidence. BJPsych Int.

[15] Bhatia S, Sethi N (2007). International community psychology: History and theories.

[16] Rabbani F, Akhtar S, Nafis J (2023). Addition of mental health to the lady health worker curriculum in Pakistan: now or never. Hum Resour Health.

[17] World Health Organization Global health expenditure database. https://apps.who.int/nha/database.

[18] The EuroQoL Group (2019). EQ5D 3l valuation, Euroqol research foundation. https://euroqol.org/eq-5d-instruments/eq-5d-3l-about/valuation.

[19] Jyani G, Sharma A, Prinja S (2022). Development of an EQ-5D Value Set for India Using an Extended Design (DEVINE) Study: The Indian 5-Level Version EQ-5D Value Set. V Health.

[20] van Hout B, Janssen MF, Feng Y-S (2012). Interim scoring for the EQ-5D-5L: mapping the EQ-5D-5L to EQ-5D-3L value sets. Value Health.

[21] Choice W (2012). Choosing interventions that are cost effective.

[22] Drummond M, Manca A, Sculpher M (2005). Increasing the generalizability of economic evaluations: recommendations for the design, analysis, and reporting of studies. Int J Technol Assess Health Care.

[23] Manca A, Rice N, Sculpher MJ (2005). Assessing generalisability by location in trial-based cost-effectiveness analysis: the use of multilevel models. Health Econ.

[24] Ramsey S, Willke R, Briggs A (2005). Good research practices for cost-effectiveness analysis alongside clinical trials: the ISPOR RCT-CEA Task Force report. Value Health.

[25] Curtis LA, Burns A (2015). Unit Costs of Health and Social Care 2015.

[26] Derakhshani N, Doshmangir L, Ahmadi A (2020). Monitoring Process Barriers and Enablers Towards Universal Health Coverage Within the Sustainable Development Goals: A Systematic Review and Content Analysis. Clinicoecon Outcomes Res.

