# Supplementary material for: Helping people with psychosis with a low-cost intervention DIALOG+: protocol for the economic evaluation in a randomised control trial in India and Pakistan
Source: BMJ Open. 2025 Jan 8;15(1):e080737. doi: 10.1136/bmjopen-2023-080737 (PMC11749519; doi:10.1136/bmjopen-2023-080737)
Supplement: Supplementary file 1 [file bmjopen-2023-080737.reviewer_comments.pdf]

PEER REVIEW HISTORY

BMJ Open publishes all reviews undertaken for accepted manuscripts. Reviewers are asked to complete a checklist review form and are provided with free text boxes to elaborate on their assessment. These free text comments are reproduced below.

ARTICLE DETAILS

Title (Provisional)

Helping people with Psychosis with a low-cost intervention DIALOG+: Protocol for the economic evaluation in a Randomized Control Trial in India and Pakistan

Authors

Malik, Ashar Muhammad; Peppl, Renata; Sajun, Sana Zehra Zehra; Qureshi, Onaiza; Priya, Krishna; Sarwar, Hufsa; Ramachandran, Padmavati; Venkatraman, Lakshmi; Evans-Lacko, Sara; Bird, Victoria Jane

VERSION 1 - REVIEW

**Reviewer**	**1**
**Name**	**Morillo , Hannah**
**Affiliation**	**King's College London Mathematics School**
**Date**	**26-Nov-2023**
**COI**	**There are no competing interests.**

To the Authors:

An economic evaluation for the study, "*Helping people with Psychosis with a low-cost intervention DIALOG+: Protocol for the economic evaluation in a Randomized Control Trial in India and Pakistan*" is a relevant undertaking. It enables a targeted approach to account the costing of the intervention outcomes of DIALOG+ as opposed to a comparator, especially in low resource settings such as in this context. The protocol provided for the general aim, scientific background, methodology of an overall economic analysis through the ICERs in cost-effectiveness analysis (CEA) and cost utility analysis (CUA), and the analysis and presentation mainly via the CHEERS. Below are some pointers to consider in your revisions:

1. Some parts of the abstract were not clearly stated in the main text, such as "Probabilistic sensitivity analysis".
2. It is suggested to be more consistent with a more formal, academic writing. There were words or phrases such as, "household's", "crucial", "piggybacked", "will help", "unblinded", "GBP/GB pounds", "to treat [skewness]", and "Psychosis" (capitalized P), that stray farther from the scientific tone and writing of the main text. Sentences such as in the conclusion, "Economic evaluation is the framework of informed priority settings." reads like an incomplete thought. In addition, sentences like "We will provide useful information to the research community on economic evaluation in clinical trials in LMICs..", "our analysis will be useful for practical purposes of the management of health facilities in the public sector..", "Our findings will help", and "will help the hospital administration to analyse.." read like a promise and/or claim

and veer away from scientific language. This article may benefit from another round of editing or two to streamline academic writing and grammatical loose ends.

3. In the introduction, it helps to further describe the connection or history behind DIALOG+ in relation to the PIECEs study. “WP2” on p.2 is an undefined term and might confuse readers.
4. Can the authors further illustrate the “weak” mental health system in India/Pakistan? (Perhaps in terms of statistics, number, or a more descriptive depiction of this observation)
5. “*We follow the guidelines on reporting the economic evaluation of healthcare programmes developed by the task force on good research practice in economic evaluation of International Society for Pharmacoeconomics and Outcomes Research and the checklist for reporting the economic evaluation recommended by Drummond and Sculpher et al 2005*” on p.3 – Authors can identify what these guidelines are and introduce them here.
6. It might help to write the equation for the cost-utility analysis when evaluate the proportion of the difference in costs of the intervention or treatment and comparator over their difference in outcomes, or in this case effectiveness of the EQ-5D-5L. Presenting an equation particularly in the *Types of Analysis* section or when the elements of the ICER equation was actually discussed on p.8 (*Analysis and reporting results*) would help the reader visualize this more clearly.
7. In the *Types of Analysis* part, “Since data will be collected on clinical effectiveness, we will also consider reporting cost-effectiveness analysis” – this sounds like an after-thought within the process. Authors can also be more intentional about evaluation the DIALOG+ via CEA *and* CUA by stating this as such. However, if the

authors are accounting for the cost-effectiveness ratio in CEA as a step in the process, they can also be more explicit in reporting this step.

8. The aim is clear: to provide an econ eval for LMICs, esp in the South Asian context. Can authors cite a similar economic evaluation in HICs? How can the results lend insight or relevance to and encourage other LMICs/Asian contexts?
9. Please include CHEERS checklist as a supplementary material. Authors may find it beneficial for the readers especially for healthcare professionals and stakeholders who are not too familiar with economic evaluation reporting standards, to include the CHEERS checklist as an appendix or supplementary material, or perhaps adding to cite a number of items in the main text.
10. In the Discussion part "The protocol described in this paper pertains to the economic evaluation component of the PIECEs RCT. It does not include methods related to data collection, data cleaning, imputation…", while the PIECEs study may account for this, in the actual economic evaluation, how do the authors propose to account for the specific estimates of the values. While ICERs were discussed and will be accounted for, these may not be enough to estimate the values within the perspectives of the decision makers involved. They might want to include this to present a more robust (i.e., to ensure reliability) economic evaluation so that it does not read as an arbitrary outcome of the result.
11. Authors may want to format the figure and table to reflect a clearer presentation of data. The figure appears a little unclear, esp. when presented as a stand-alone figure. A descriptive title or short caption may communicate a clearer message.
12. Please use more supporting statements in the conclusion.
13. The results and discussion were very specific in the ratios and anticipated costing, among others. Perhaps this can also be reflected in the aim of the paper, potentially adding specific objectives you would like to explore or are anticipating in your economic evaluation.

Very best wishes.

**Reviewer**	**2**
**Name**	**Shields, Gemma**
**Affiliation**	**University of Manchester, Centre for Health Economics**
**Date**	**11-Jun-2024**
**COI**	**None**

This is a clear protocol, I only have some minor comments:

Does the comparator represent current standard of care? Or is dialog on top of treatment as usual?

Are all of the resources associated with running an RCT relevant for inclusion in the economic evaluation? If rolled out in the real-world, would these costs be different?

There is quite a small sample of TMS data, why is it only a sample?

Are the authors conducting their own mapping study for the EQ-5D-5L in Pakistan?

Could a value set from another country be used?

I'm not clear why a pooled analysis for both countries is helpful or would be meaningful for anyone.

What threshold will be used to interpret cost-effectiveness in each country?

What software will be used?

Will healthcare costs be reported by participants in data collection (or rather will resource use be collected, with national unit costs used)?

In line with CHEERS2, have the authors considered patient engagement in their design? Or accounting for distributional effects?

VERSION 1 - AUTHOR RESPONSE

Dear Reviewer,

We appreciate you contribution to help us improve the manuscript. We have updated the manuscript in the light of your views and have provided point by point replies to your concerns in the text below in Red italic font.

Reviewer 1

1. Some parts of the abstract were not clearly stated in the main text, such as

"Probabilistic sensitivity analysis".

We have added this type of sensitivity analysis in the main text in section on Characterizing uncertainty.

2. It is suggested to be more consistent with a more formal, academic writing. There were words or phrases such as, "household's", "crucial", "piggybacked", "will help", "unblinded", "GBP/GB pounds", "to treat [skewness]", and "Psychosis" (capitalized P), that stray farther from the scientific tone and writing of the main text. Sentences such as in the conclusion, "Economic evaluation is the framework of informed priority settings." reads like an incomplete thought. In addition, sentences like "We will provide useful information to the research community on economic evaluation in clinical trials in LMICs..", "our analysis will be useful for practical purposes of the management of health facilities in the public sector..", "Our findings will help", and "will help the hospital administration to analyse.." read like a promise and/or claim and veer away from scientific language. This article may benefit from another round of editing or two to streamline academic writing and grammatical loose ends.

We have addressed these issues to ensure consistency and objectively throughout the paper. Please refer to the edited version of the paper.

3. In the introduction, it helps to further describe the connection or history behind DIALOG+ in relation to the PIECEs study. “WP2” on p.2 is an undefined term and might confuse readers.

We have added description of DIALOG+ in paragraph 3 of introduction section.

4. Can the authors further illustrate the “weak” mental health system in India/Pakistan? (Perhaps in terms of statistics, number, or a more descriptive depiction of this observation)

We have added more contents at the end of paragraph 1 of the introduction section to strengthen argument of weak healthcare system in LMICs such as India and Pakistan.

5. “We follow the guidelines on reporting the economic evaluation of healthcare programmes developed by the task force on good research practice in economic evaluation of International Society for Pharmacoeconomics and Outcomes Research and the checklist for reporting the economic evaluation recommended by Drummond and Sculpher et al 2005” on p.3 – Authors can identify what these guidelines are and introduce them here.

We provide more details for CHEERs and Drummond’s checklist at the end of paragraph 1 of page 5.

6. It might help to write the equation for the cost-utility analysis when evaluate the proportion of the difference in costs of the intervention or treatment and comparator over their difference in outcomes, or in this case effectiveness of the EQ-5D-5L. Presenting an equation particularly in the Types of Analysis section or when the elements of the ICER equation was actually discussed on p.8 (Analysis and reporting results) would help the reader visualize this more clearly.

We have provided equation of ICERs at paragraph 2 of page 7.

7. In the Types of Analysis part, “Since data will be collected on clinical effectiveness, we will also consider reporting cost-effectiveness analysis” – this sounds like an after-thought within the process. Authors can also be more intentional about evaluation the DIALOG+ via CEA and CUA by stating this as such. However, if the authors are accounting for the cost-effectiveness ratio in CEA as a step in the process, they can also be more explicit in reporting this step.

We revamped section on type of analysis at page 5 with more clarity to describe difference in CEA and CUA and their respective uses.

8. The aim is clear: to provide an econ eval for LMICs, esp in the South Asian context. Can authors cite a similar economic evaluation in HICs? How can the results lend insight or relevance to and encourage other LMICs/Asian contexts?

We have added reference (reference number 6) of similar kind of analysis testing DIALOG+ in high income countries and referred this in the text.

9. Please include CHEERS checklist as a supplementary material. Authors may find it beneficial for the readers especially for healthcare professionals and stakeholders who are not too familiar with economic evaluation reporting standards, to include the CHEERS checklist as an appendix or supplementary material, or perhaps adding to cite a number of items in the main text.

We have provided the references number 12 and 13 to these documents. The CHEERS is available in open access and published Value in Health journals while the Drummond Check list is copyrighted material of Oxford University Press for which we may need special permission.

10. In the Discussion part “The protocol described in this paper pertains to the economic evaluation component of the PIECEs RCT. It does not include methods related to data collection, data cleaning, imputation…”, while the PIECEs study may account for this, in the actual economic evaluation, how do the authors propose to account for the specific estimates of the values. While ICERs were discussed and will be accounted for, these may not be enough to estimate the values within the perspectives of the decision makers involved. They might want to include this to present a more robust (i.e., to ensure reliability) economic evaluation so that it does not read as an arbitrary outcome of the result.

We mentioned that the study is piggy backed to the RCT and protocol of RCT and statistical analysis plan are published separately. We mentioned this as a limitation of our study at the end of the discussion part which included comparison arms of RCT. This will not undermine the robustness of analysis since it is an embedded limitation of the RCT.

11. Authors may want to format the figure and table to reflect a clearer presentation of data. The figure appears a little unclear, esp. when presented as a stand-alone figure. A descriptive title or short caption may communicate a clearer message.

We have added notes to figure and table to make them self-sufficient.

12. Please use more supporting statements in the conclusion.

Conclusion is updated please.

13. The results and discussion were very specific in the ratios and anticipated costing, among others. Perhaps this can also be reflected in the aim of the paper, potentially adding specific objectives, you would like to explore or are anticipating in your economic evaluation.

We have revised the section on aims and objectives and added some specific features of the analysis.

Thank you

Reviewer 2

Does the comparator represent current standard of care? Or is dialog on top of treatment as usual?

Due to the ethical considerations the PIECEs RCT is designed to include an active control instead of usual management of Psychosis in India and Pakistan. We therefore must follow the same comparator in economics evaluation.

Are all the resources associated with running an RCT relevant for inclusion in the economic evaluation? If rolled out in the real-world, would these costs be different?

Paragraph 2 page 8 provides the resources provided by the RCT to administer DIALOG+. These resources are estimated using the real-world settings of the field sites of RCT in both countries. As such we can assume that the cost will represent real world settings if the intervention is rolled out.

There is quite a small sample of TMS data, why is it only a sample?

TMS is conducted on a sub sample of the RCT. This was so because some data was already collected prior to the start of the TMS.

Are the authors conducting their own mapping study for the EQ-5D-5L in Pakistan?

Could a value set from another country be used?

Value sets of EQ5D are being developed in both the countries and will be used to estimate QALYs. We are in contact with EUROQOL group to access the values sets of EQ5D for India and Pakistan.

I’m not clear why a pooled analysis for both countries is helpful or would be meaningful for anyone.

The results of RCT will be published for both the countries and will include economic evaluation as well. Moreover, pooled analysis is relevant to submitting report to the funding agency that has funded this research in both countries.

What threshold will be used to interpret cost-effectiveness in each country?

We have added threshold analysis in the methods section at paragraph 1 of page 10.

What software will be used?

We will used Microsoft Excel and STATA version 16 for windows for analysis of data. We have mentioned these details at last paragraph of page 10.

Will healthcare costs be reported by participants in data collection (or rather will resource use be collected, with national unit costs used)?

The cost of caregiver and patients will be used. Pease refer to the table for details on costs centre defined in the analysis and sources of data.

In line with CHEERS2, have the authors considered patient engagement in their design? Or accounting for distributional effects?

We did not include distribution analysis since the patient population of the RCT is mainly from low-income families. The healthcare providers are from public, and charity sectors which mainly serve patients from low and low middle socio-economic status.

Thank you
